# A lactate-targeted resuscitation strategy may be associated with higher mortality in patients with septic shock and normal capillary refill time: a post hoc analysis of the ANDROMEDA-SHOCK study

**DOI:** 10.1186/s13613-020-00732-1

**Published:** 2020-08-26

**Authors:** Eduardo Kattan, Glenn Hernández, Gustavo Ospina-Tascón, Emilio Daniel Valenzuela, Jan Bakker, Ricardo Castro, The ANDROMEDA-SHOCK Study Investigators and the Latin America Intensive Care Network (LIVEN)

**Affiliations:** 1 Departamento de Medicina Intensiva, Facultad de Medicina, Pontificia Universidad Católica de Chile, Santiago, 362 Chile; 2 Department of Intensive Care Medicine, Fundación Valle del Lili, Universidad ICESI, Carrera 98 # 18-49, Cali, Colombia; 3 Division of Pulmonary, Allergy, and Critical Care Medicine, Columbia University Medical Center, 630 W 168th St, New York, USA; 4 Department Intensive Care Adults, Erasmus MC University Medical Center, Rotterdam, CA The Netherlands; 5 Division of Pulmonary, and Critical Care Medicine, New York University-Langone, New York, USA

**Keywords:** Septic shock, Sepsis, Early resuscitation, Capillary refill time, Lactate, Peripheral perfusion

## Abstract

**Background:**

Capillary refill time (CRT) may improve more rapidly than lactate in response to increments in systemic flow. Therefore, it can be assessed more frequently during septic shock (SS) resuscitation. Hyperlactatemia, in contrast, exhibits a slower recovery in SS survivors, probably explained by the delayed resolution of non-hypoperfusion-related sources. Thus, targeting lactate normalization may be associated with impaired outcomes. The ANDROMEDA-SHOCK trial compared CRT- versus lactate-targeted resuscitation in early SS. CRT-targeted resuscitation associated with lower mortality and organ dysfunction; mechanisms were not investigated. CRT was assessed every 30 min and lactate every 2 h during the 8-h intervention period, allowing a first comparison between groups at 2 h (T2). Our primary aim was to determine if SS patients evolving with normal CRT at T2 after randomization (T0) exhibited a higher mortality and organ dysfunction when allocated to the LT arm than when randomized to the CRT arm. Our secondary aim was to determine if those patients with normal CRT at T2 had received more therapeutic interventions when randomized to the LT arm. To address these issues, we performed a post hoc analysis of the ANDROMEDA-SHOCK dataset.

**Results:**

Patients randomized to the lactate arm at T0, evolving with normal CRT at T2 exhibited significantly higher mortality than patients with normal CRT at T2 initially allocated to CRT (40 vs 23%, *p* = 0.009). These results replicated at T8 and T24. LT arm received significantly more resuscitative interventions (fluid boluses: 1000[500–2000] vs. 500[0–1500], *p* = 0.004; norepinephrine test in previously hypertensive patients: 43 (35) vs. 19 (19), *p* = 0.001; and inodilators: 16 (13) vs. 3 (3), *p* = 0.003). A multivariate logistic regression of patients with normal CRT at T2, including APACHE-II, baseline lactate, cumulative fluids administered since emergency admission, source of infection, and randomization group) confirmed that allocation to LT group was a statistically significant determinant of 28-day mortality (OR 3.3; 95%CI[1.5–7.1]); *p* = 0.003).

**Conclusions:**

Septic shock patients with normal CRT at baseline received more therapeutic interventions and presented more organ dysfunction when allocated to the lactate group. This could associate with worse outcomes.

## Background

ANDROMEDA-SHOCK was a randomized controlled trial comparing capillary refill time (CRT)- versus lactate-targeted (LT) resuscitation in early septic shock [1], that suggested a lower mortality, and demonstrated significantly less organ dysfunction and treatment intensity in the CRT group. A subsequent Bayesian post hoc analysis supported the survival benefit of a CRT-targeted resuscitation [2].

CRT is a flow-sensitive variable that may improve rapidly after an increase in systemic blood flow [3–6]. Therefore, it can be assessed more frequently, and resuscitation could be stopped earlier than when a lactate endpoint is pursued; indeed, lactate exhibits a slow kinetics of recovery even in septic shock survivors [7, 8]. CRT was the first perfusion variable to reach a significant improvement 2 h after ICU-based resuscitation [3], and 70% of septic shock survivors exhibited a normal CRT at 2 h[7]. A normal CRT at 2 h after initial or advanced fluid resuscitation was associated with less than 15% mortality risk [3, 4]. Moreover, in a previous pilot study, fluid resuscitation could be safely withheld in septic shock patients with normalized peripheral perfusion, a fact that was associated with less organ dysfunction [9]. In ANDROMEDA-SHOCK, CRT was assessed every 30 min and lactate every 2 h during the intervention period of 8 h [10, 11]. Accordingly, and considering the published evidence, the first time-point where the impact of resuscitation could be compared between study arms was as early as at 2 h (T2). Besides, the status of CRT at 2 h could have prognostic value and aid to take decisions on further resuscitation [3, 4].

The lower mortality and less organ dysfunction observed in septic shock patients randomized to CRT-targeted resuscitation is significant and deserves further exploration. Eventually, a normal CRT in septic shock patients with hyperlactatemia signals a predominant non-hypoperfusion-related source for lactate where no subsequent and potentially deleterious resuscitation is probably required [5]. In fact, there are several non-hypoxia related alternative explanations for persistent hyperlactatemia [12]. Stress-related hyperlactatemia is triggered by the neurohumoral response to sepsis that generates aerobic lactate production in skeletal muscles via beta-2 epinephrine stimulation. This lactate acts as a metabolic shuttle providing energy to other organs, and this process can be blocked by specific interventions [13, 14]. On the other hand, Tapia et al. demonstrated a severe impairment in exogenous lactate clearance very early after experimental endotoxic shock induction, not related to liver hypoperfusion as demonstrated by several techniques [15]. The authors suggested a metabolic blockade as a potential explanation for this finding.

Our hypothesis was that in SEPSIS-3 septic shock patients evolving with normal CRT at 2 h, the T0 randomization to the LT arm was associated with a higher mortality compared to patients randomized to the CRT arm. Our primary aim was to determine if septic shock patients evolving with normal CRT at T2 exhibited a higher mortality and organ dysfunction after being randomized to the LT arm at T0 than when randomized to the CRT arm. Our secondary aim was to determine if those septic shock patients evolving with normal CRT at T2 received more therapeutic interventions when randomized to the LT arm at T0 than when randomized to the CRT arm.

To address this issue, we performed a post hoc analysis of the ANDROMEDA-SHOCK dataset.

## Materials and methods

### Data collection and processing

The ANDROMEDA-SHOCK trial patients fulfilled the SEPSIS-3 criteria [16] (i.e., presence of suspected infection accompanying life-threatening organ dysfunction, requirement use of vasopressors to maintain mean arterial pressure (MAP) > 65 mmHg, and lactate levels > 2 mmol/L). Conversely, CRT status was not incorporated as inclusion criteria.

The detailed protocol of ANDROMEDA-SHOCK trial including the stepwise interventional procedures can be found elsewhere [10, 11]. Briefly, during the 8-h intervention period, the goal for the CRT arm was to normalize CRT (normal value ≤ 3 s as assessed with a standardized technique), whereas the goal for the LT arm was to normalize (normal values ≤ 2 mmol/L) or to decrease lactate levels by 20% every 2 h. Following initial fluid resuscitation and norepinephrine (NE) to reach and maintain a mean arterial pressure (MAP) ≥ 65 mm Hg, both groups were managed with an identical sequential protocolized approach. In both groups, the first step was assessment of fluid responsiveness, followed by fluid challenges with 500 ml of crystalloids every 30 min in fluid-responders until the goal was achieved or a central venous pressure safety limit was reached, or the patient became fluid unresponsive, whichever came first. As a second step, a vasopressor test was performed in previously chronic hypertensive patients in whom targets were not achieved with fluids. NE was transiently increased until reaching a MAP of 80 to 85 mmHg followed by a reassessment of CRT or lactate after one or two hours, respectively. If the goal was met, this MAP level was maintained throughout the intervention period. The third step consisted of the use of low dose dobutamine or milrinone. Patients were again reassessed after one or two hours in the CRT and LT group, respectively. If the endpoints were still not met, or a safety issue arose, the inodilator was discontinued.

We examined the relationship between CRT status at 2 h after randomization (T2) with clinical characteristics, interventions, and outcomes, including mortality at 28 days for the whole cohort and according to the study group allocation. Then, we analyzed the impact of the randomization arm in both groups in patients with normal CRT at T2. Finally, we performed the same analysis in lactate target achievers (normalization or 20% decrease) at T2.

The main outcome of this study was all-cause mortality at 28 days. Secondary outcomes included severity scores, like the Acute Physiology and Chronic Health Evaluation (APACHE) II score [17], Charlson index [18], and daily Sequential Organ Failure Assessment (SOFA) [19]. Severity of hemodynamic derangements and intensity of therapy was assessed through the evolution of perfusion variables along time, including lactate, CRT, central venous oxygen saturation (ScvO2), central venous-to-arterial pCO2 gradient (P(cv‑a)CO2) or dCO2), dosage of vasopressors, total amount of fluid boluses and fluid balance.

As therapeutic interventions were guided by a predefined protocol that included a stepwise approach to resolve hypoperfusion, we developed a composite outcome that included each protocol-driven resuscitative step taken by attending physicians. Every 500 ml of fluid bolus, vasopressor test, or inodilator test was considered a resuscitative action with a numerical value of 1, and the cumulative number of actions were summed-up for each patient. Finally, other clinically relevant outcomes were registered, like need for mechanical ventilation (MV), renal replacement therapy (RRT), plus ICU and hospital length of stay.

For variables with non-normal distribution, non-parametric tests were used. Accordingly, descriptive statistics are shown as medians [interquartile range 25–75] or percentages (%). Mann–Whitney U, Kruskal–Wallis, Chi-square, and Fisher’s exact, were used when appropriate. Data was analyzed with Minitab v17 (Minitab Inc, State College, PA) and Graphpad Prism (Graphpad Softwares, La Joya, CA) softwares. Two-tailed *p* value of < 0.05 was considered as statistically significant.

## Results

ANDROMEDA-SHOCK study included 424 patients whose main characteristics are presented in Additional File 1. As previously reported [1], 378 patients had available data at 2 h, so this subset was used for T2 data analysis.

### Impact of normal CRT at T2

**Fig. 1:**
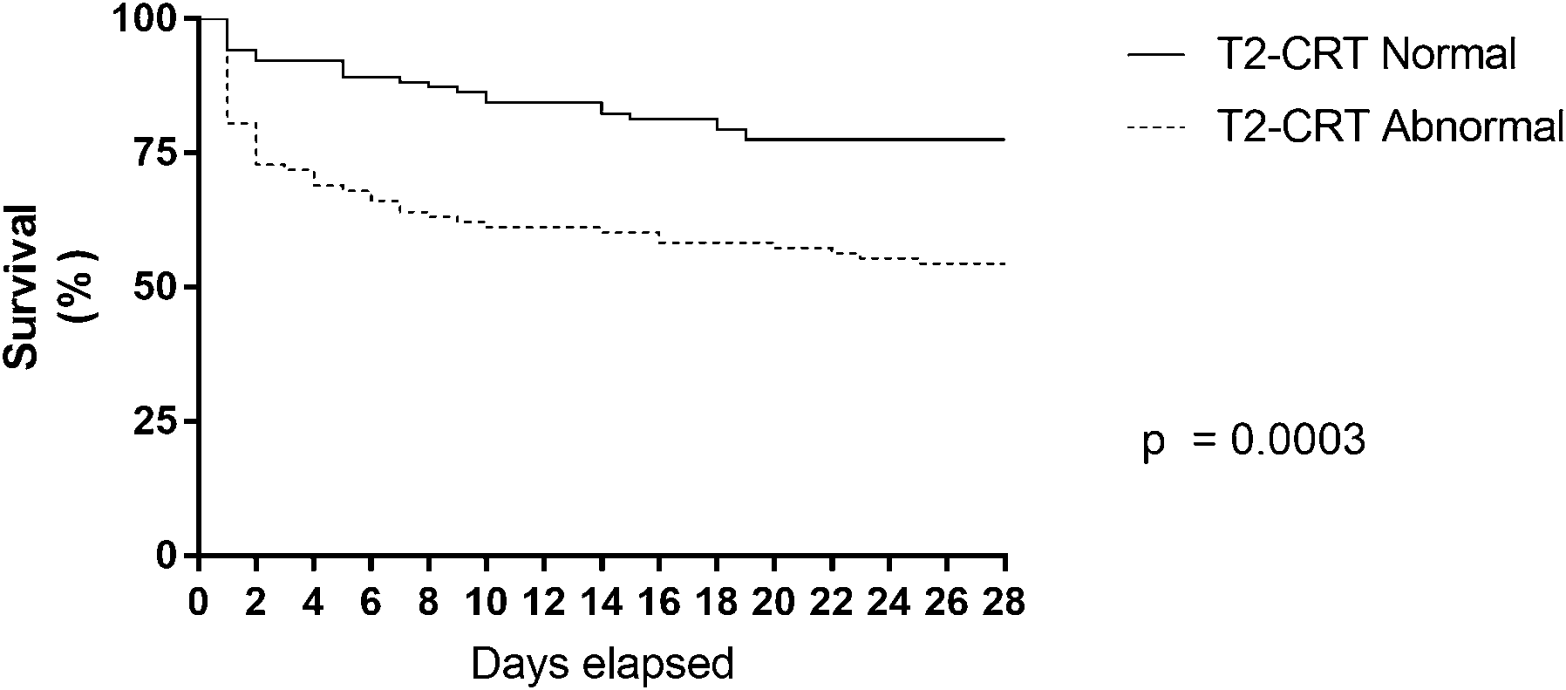
Kaplan–Meier estimates of the 28-day survival rate according to CRT status at 2 h

After two hours of protocolized ICU-resuscitation, 49% of patients (184/378) progressed with a normal CRT (Table 1). Regardless of the study group allocation, those patients received less resuscitative interventions, evolved with lower SOFA score at 24 h (7 [4–10] vs 10 [6–13], *p* = 0.0001), and exhibited a lower mortality at 28 days (30% vs 46%, *p* = 0.002) compared to patients with abnormal CRT at T2 (Table 1, Fig. 1, Additional File 2).

**Table 1:** Baseline characteristics and clinical outcomes of septic shock patients with normal versus abnormal capillary refill time at 2 h from inclusion

	CRT normal	CRT abnormal	P
Number of patients	184 (48.7)	194 (51.3)	
Age (years)	63 [60–80]	69 [56–77]	0.0001
Sex (female)	88 (47.8)	93 (47.9)	0.9
APACHE score	20 [14–25]	24 [18–29]	0.0001
SOFA score	9 [7–11]	10 [8–13]	0.0001
Charlson index	3 [1–5]	3 [1–5]	0.9
Randomization arm	LT: 82 (45)	LT: 92 (48)	0.5
	CRT-T: 102 (55)	CRT-T:102 (52)	
Sepsis origin	Abdominal 64 (35)	Abdominal: 74 (38)	0.3
	Pulmonary 55 (30)	Pulmonary: 52 (27)	
	Urinary 37 (20)	Urinary: 45 (23)	
	Other 28 (15)	Other: 23 (12)	
MAP (mmHg)	68 [64–78]	64 [56–73]	0.0001
CVP (mmHg)	9 [5–13]	9 [7–13]	0.3
Fluids administered before ICU admission (ml)	2000 [1350–2907]	2000 [1200–2500]	0.13
Fluid responsiveness positive state	98 (53)	120 (62)	0.09
Fluid administered in boluses between 0 and 8 h (ml)	1000 [0–1500]	1500 [500–2500]	0.001
Fluid balance at 8 h (ml)	1244 [480–2136]	1842 [977–2977]	0.001
Norepinephrine dose (mcg/kg/min)	0.17 [0.1–0.3]	0.26 [0.14–0.43]	0.0001
Lactate (mmol/L)	3.3 [2.6–4.4]	4 [2.9–6.5]	0.0001
CRT (s)	4 [2–5]	6 [5–7]	0.0001
ScvO2 (%)	73 [65–80]	72 [62–78]	0.36
Delta pCO2(v-a)	7 [4–10]	7 [5–10]	0.3
SOFA at 24 h	7 [4–10]	10 [6–13]	0.0001
Renal replacement therapy	27 (15)	39 (20)	0.16
Mechanical ventilation	123 (67)	163 (84)	0.001
ICU length of stay (days)	6 [3–11]	6 [2–10]	0.45
28-day mortality	56 (30)	89 (46)	0.002

Data are presented as median [IQR 25–75] or count (percentage)

Data at 8 and 24 h are reported for clarification purposes

*APACHE II* Acute Physiology And Chronic Health Evaluation II, *SOFA* Sequential organ failure Assessment score, *LT* lactate-targeted group, *CRT-T* CRT targeted group, *ICU* intensive care unit, *MAP* mean arterial pressure, *CVP* central venous pressure, *CRT* capillary refill time, *ScvO2* central venous oxygen saturation, *Delta pCO2(v-a)* difference between central venous carbon dioxide pressure and arterial carbon dioxide pressure

### Normal CRT and randomization arm at T2

**Fig. 2:**
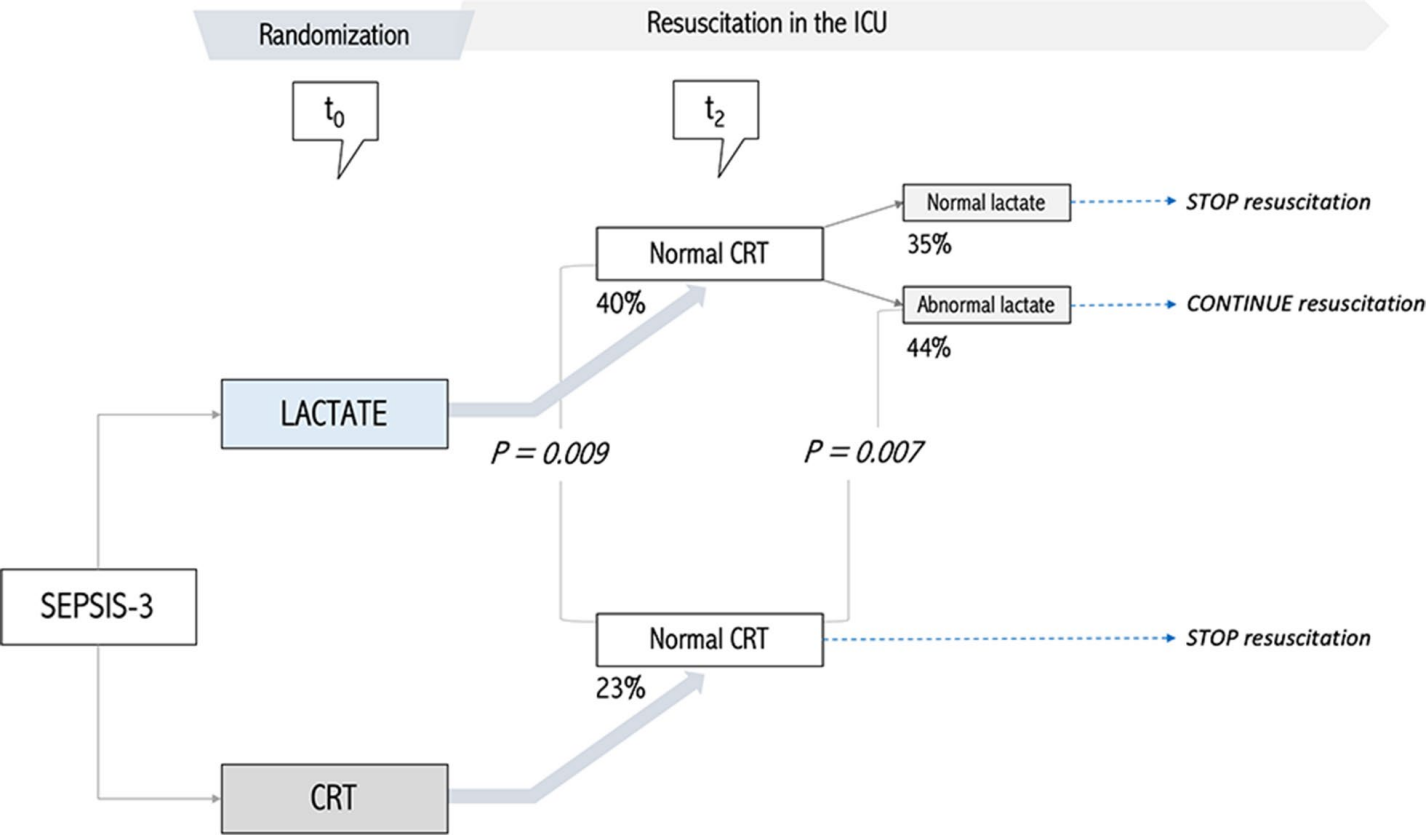
Patients distribution at 0 and 2 h after ICU admission and associated outcome according to randomization group, under CRT state perspective. Percentages refer to 28-day mortality. All patients started with hyperlactatemia. Lactate arm pursued lactate normalization or significant lowering, irrespective of CRT state. CRT arm pursued CRT normalization

**Fig. 3:**
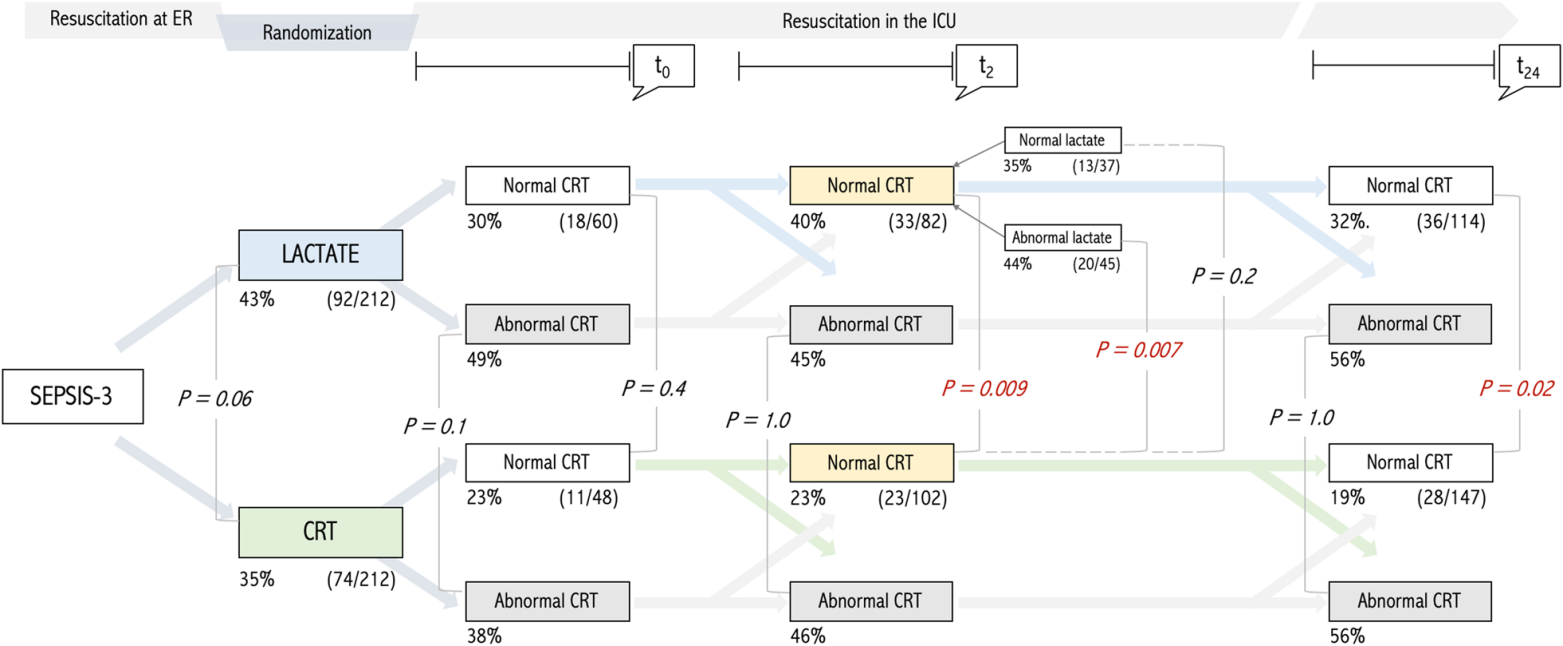
Patients distribution at 0, 2, 8 and 24 h and associated outcome according to randomization group, under CRT perspective. Percentages refer to 28-day mortality. All patients started with hyperlactatemia. Lactate arm pursued lactate normalization or significant lowering, irrespective of CRT state. CRT arm pursued CRT normalization

Patients with normal CRT at T2 randomized to the LT arm evolved exhibiting a significantly higher mortality than patients with normal CRT at T2 that were randomized to the CRT group (40 vs 23%, *p* = 0.009) (Table 2, Figs. 2 and 3). They also received significantly more supportive therapies and resuscitative interventions (fluids, vasopressor and inodilator tests) at the end of the intervention period (Table 2). This difference was mainly driven by patients in the LT group that had not reached their resuscitation endpoint at T2 (44 vs 23%, *p* = 0.007) (Table 2, Figs. 2 and 3) and was maintained at protocol end at T8 (35 vs 34%, *p* = 0.055) and at T24 (32 vs 19%, *p* = 0.02) (Fig. 3). Figure 3 also shows differences between patients who maintained abnormal CRT across timepoints, according to the randomization group.

**Table 2:** Clinical and interventions comparison between CRT responders at T2, according to study group

Original study arm	CRT Normal at 2 h (*n* = 184)		*P*
	CRT	Lactate	
Number of patients	102 (55)	82 (45)	
Age (years)	62 [46–72]	65 [49–74]	0.23
Sex	Female 49 (48)	Female 39 (48)	0.90
	Male 53 (52)	Male 43 (52)	
APACHE II score	20 [14–26]	19 [16–24]	0.96
SOFA score	9 [7–11]	9 [7–11]	0.63
Charlson Index	3 [1–5]	3 [1–5]	0.76
Sepsis origin *n* (%)	Urinary 17 (17)	Urinary 20 (24)	0.36
	Pulmonary 37 (36)	Pulmonary 18 (22)	
	Abdominal 33 (33)	Abdominal 31 (37)	
	Other 14 (14)	Other 14 (17)	
MAP (mmHg)	70 [65–83]	67 [62–75]	0.01
CVP (mmHg)	9 [6–13]	9 [5–14]	0.70
Pre-protocol fluids (ml)	2000 [1238–2850]	2050 [1500–2957]	0.15
NE dose (mcg/kg/min)	0.18 [0.1–0.31]	0.15 [0.1–0.3]	0.60
Baseline lactate (mmol/L)	3.3 [2.6–4.4]	3.2 [2.7–4.3]	0.73
Baseline CRT (s)	4 [2–5]	3 [2–4]	0.15
Baseline mottling score	0 [0–1]	0 [0–1]	0.60
Baseline ScvO_2_ (%)	74 [68–79]	71 [62–80]	0.40
Baseline dCO_2_(v-a)	7 [4–9]	7 [5–10]	0.70
Fluid bolus 0–8 h (ml)	500 [0–1500]	1000 [500–2000]	0.004
Fluid balance 8 h (ml)	1090 [319–2000]	1360 [559–2401]	0.038
Vasopressor test	19 (19)	35 (43)	0.001
Inodilator test	3 (3)	13 (16)	0.003
Resuscitative interventions	1.25 [0.5–3]	3 [1.8–4.2]	0.001
SOFA 24 h	7 [4–10]	8 [5–11]	0.11
dSOFA 0-24 h	2 [0–4]	1 [−1–3]	0.004
RRT	11 (11)	16 (19)	0.1
MV	66 (65)	57 (70)	0.50
ICU length of stay (days)	6 [3–12]	6 [3–11]	0.60
28-day mortality	23 (23)	33 (40)	0.009

Data are presented as median [IQR 25–75] or count (percentage)

*CRT* capillary refill time, *APACHE II* Acute Physiology And Chronic Health Evaluation II, *SOFA* Sequential organ failure Assessment score, *ICU* intensive care unit, *MAP* mean arterial pressure, *CVP* central venous pressure, *NE* norepinephrine, *ScvO2* central venous oxygen saturation, *Delta pCO2(v-a)* difference between central venous carbon dioxide pressure and arterial carbon dioxide pressure, *dSOFA* delta SOFA, *RRT* renal replacement therapy, *MV* mechanical ventilation

Moreover, a multivariate logistic regression of patients with normal CRT at T2, including clinically relevant variables at baseline (APACHE-II, baseline lactate, fluids administered since emergency admission until T2, source of infection, and randomization group) confirmed that allocation to the LT study group was a statistically significant determinant of 28 day mortality (OR 3.3; 95%CI[1.5-7.1]); *p* = 0.003). In the same regression, APACHE II (OR 1.1; 95%CI[1.0-1.2]); *p* < 0.001) and pulmonary source of infection (OR 4.2; 95%CI[1.7-10.6]; *p* = 0.002) impacted significantly on mortality at 28 days.

### Lactate endpoint achievement and randomization arm at T2

Patients with normal or 20% reduction on lactate at T2, exhibited no statistically significant difference in mortality (25 vs 33%, *p* = 0.4), APACHE-II score (20 [17–27] vs 19 [16–25], *p* = 0.6), and resuscitative interventions (2 [1–4] vs 2 [1–4], *p* = 0.85), when compared by randomization arm.

## Discussion

Our results suggest that septic shock patients exhibiting normal peripheral perfusion early after starting protocolized resuscitation could present worse outcomes when treated with a lactate-targeted strategy. In fact, in this post hoc analysis, these patients presented higher mortality, received more interventions such as fluids or vasoactive agents, and evolved with a slower decrease in organ dysfunction scores when compared to those treated with a CRT-targeted resuscitation strategy.

Current Surviving Sepsis Campaign (SSC) guidelines recommend targeting normalization of lactate during septic shock resuscitation [20]. However, this recommendation does not consider that there are multiple pathogenic mechanisms involved in persistent hyperlactatemia, and that a relatively high proportion appears to be non-hypoperfusion-related [12, 21]. A previous retrospective proof-of-concept study showed that septic shock patients with hyperlactatemia but without a hypoperfusion context, as demonstrated by concomitant normal ScvO_2_, dCO_2_ or CRT, tended to exhibit lower mortality and to require less therapeutic interventions [5]. In this sense, our results confirm the safety of withholding further resuscitation in septic shock patients with normal CRT. Moreover, and on the contrary, pursuing lactate as a therapeutic target in septic shock patients without a hypoperfusion context appears as deleterious, as this practice was associated with more interventions and higher mortality.

Septic shock patients present with hypotension and hypoperfusion, and are resuscitated in general, with rapid fluid loading and goal-directed endpoints [20, 22]. After the acute period, however, they frequently display net positive fluid balance [22, 23] and although fluid administration practices are highly variable worldwide [24], fluid overload is still a clinical problem [25]. The importance of fluid overload and fluid balance cannot be underestimated since it is a key determinant of higher morbidity and mortality in critically ill patients [22, 25–30]. According to our results, it seems that after very early protocolized resuscitation, the selected target determines the intensity of further resuscitation. Indeed, patients with normal CRT at 2 h but randomized to LT resuscitation received more fluid boluses.

In addition, per design, these patients were subjected to more protocol-loop routing and supportive therapies, eventually increasing the burden of over-resuscitation [27, 30–32]. It may be highlighted that patients randomized to the LT arm evolving with normal CRT were also exposed to more vasopressor and inodilator tests to achieve the lactate’s goal. Indeed, although previously hypertensive patients may benefit from higher MAP goals, the obligatory increase in NE dose to achieve this level has been associated with increased risk of arrhythmias [33]. The negative impact of excessive catecholamine support in septic shock has been suggested [34] and sparing of these drugs may have also contributed to the improved outcome in the CRT arm. This possible explanation should be clarified by further studies.

A cautionary note must be introduced here since at T0, patients with normal CRT randomized to the LT arm did not demonstrate overall a significantly higher mortality compared to those randomized to CRT. This may argue against the concept of the excess mortality related to an excess of resuscitative interventions, as patients in CRT with normal CRT should have received less interventions than patients with normal CRT in high lactate group. Also, the normal CRT group in the LT arm increased their mortality from 30 to 40% from T0 to T2 probably due to patients with abnormal CRT at baseline who normalized their CRT. Per protocol, these patients should have received some resuscitative interventions, even if they had been randomized to the CRT arm instead of the LT one. This finding may argue against the postulate that some excess in mortality was undoubtedly related to excess on interventions in the LT group, since in both groups they should have been similar. Other factors may have played a role, as randomization was not stratified to CRT values at baseline and subgroups with normal CRT in the LT arm may have been more severe than the subgroup with normal CRT in the CRT group.

The present study presents some insights in trying to understand the outcome differences suggested by the original ANDROMEDA-SHOCK trial report [1] and the Bayesian reanalysis of the same data [2]. Peripheral perfusion normalization may be a better resuscitation endpoint than lactate. Indeed, as patients did not differ on baseline demographics, previous fluids administration, sepsis sources or severity indices, the randomization to the group pursuing a lactate target could have determined a higher risk of death.

The clinical implications of these findings can only be expressed as hypothesis-generating ideas at this point: First, the selected resuscitation target may strongly influence the intensity of treatment with fluids or vasoactive agents since potential targets such as CRT or lactate are not equivalent in this aspect. Second, our results confirm the findings of a previous pilot study concerning the safety of withholding fluid resuscitation in septic shock patients with normal CRT [9], even though they had not cleared hyperlactatemia. Third, our data also validate the concept of non-hypoperfusion-related hyperlactatemia as suggested by previous observational studies [5, 7]. This condition might represent a less severe sepsis-related acute circulatory dysfunction that could be treated more conservatively. Fourth, pursuing lactate as a target without considering the state of peripheral perfusion might be potentially deleterious. In consequence, further resuscitation steps should be cautiously considered and on an individual basis in patients with septic shock that have already normalized CRT. Finally, the design of future randomized controlled studies on the best resuscitation target for septic shock should consider potential clinical phenotypes derived from a multimodal perfusion monitoring, a fact that deserves further research.

## Limitations

This study has some limitations. First, it is a post hoc analysis of an original study with a different design. Therefore, the suggestion that septic shock patients with a normal CRT could have a higher mortality when pursuing lactate normalization as a target is only hypothesis-generating. Second, CRT is not a perfect perfusion marker tool since there is inter-rater variability [35] that demands training and standardization. It could be difficult to apply in some clinical scenarios as hypothermia, surgery, vasculitis, etc. Novel techniques to further standardize CRT measurement have been recently proposed [36, 37] and should be tested in proper scenarios.

## Conclusions

Septic shock patients with normal CRT at baseline received more therapeutic interventions and presented more organ dysfunction when allocated to the lactate group. This could associate with worse outcomes.

## **Supplementary information**

**Additional File 1:** Baseline characteristics and clinical outcomes of septic shock patients at ANDROMEDA-SHOCK protocol inclusion. Data at 8-hours are reported for clarification purposes.

**Additional File 2:** Demographic, perfusion and hemodynamic characteristics and clinical outcomes of patients according to CRT status at T0 and T2.

**Additional File 3:** Clinical and interventions comparison between CRT non-responders at T2, according to study group.

## Abbreviations

CRT: Capillary refill time
LT: Lactate-targeted
ICU: Intensive care unit
MAP: Mean arterial pressure
T0: 0-hours
T2: 2-hours
APACHE: Acute Physiology and Chronic Health Evaluation
SOFA: Sequential Organ Failure Assessment
ScvO2: Central venous oxygen saturation
delta pCO2: Central venous–arterial pCO2 gradients
MV: Mechanical ventilation
RRT: Renal replacement therapy
SSC: Surviving Sepsis Campaign
