# Supplementary material for: A lactate-targeted resuscitation strategy may be associated with higher mortality in patients with septic shock and normal capillary refill time: a post hoc analysis of the ANDROMEDA-SHOCK study
Source: Ann Intensive Care. 2020 Aug 26;10:114. doi: 10.1186/s13613-020-00732-1 (PMC7450018; doi:10.1186/s13613-020-00732-1)
Supplement: Supplementary file 1 — Additional File 1: Baseline characteristics and clinical outcomes of septic shock patients at ANDROMEDA-SHOCK protocol inclusion. Data at 8-hours are reported for clarification purposes. [file 13613_2020_732_MOESM1_ESM.docx]

**Additional File 1:** Baseline characteristics and clinical outcomes of septic shock patients at ANDROMEDA-SHOCK protocol inclusion. Data at 8-hours are reported for clarification purposes.

| Variable |  |
| --- | --- |
| Age (years) | 66 [52-76] |
| Sex (female) | 198 (47) |
| APACHE score | 21 [16-28] |
| SOFA score | 10 [7-12] |
| Charlson index | 3 [1-5] |
| Sepsis origin | Abdominal 149 (35.1)  Pulmonary 128 (30.2)  Urinary 87 (20.5)  Other 60 (14.2) |
| MAP (mmHg) | 66 [60-76] |
| CVP (mmHg) | 9 [6-13] |
| Fluids administered before  ICU admission (ml) | 2000 [1200-2800] |
| Fluid responsiveness positive state | 242 (57) |
| Fluid administered in boluses  between 0-8 hours (ml)* | 1000 [400-2000] |
| Fluid balance at 8-hours (ml)* | 1448 [603-2483] |
| Norepinephrine dose (mcg/kg/min) | 0.21 [0.1-0.4] |
| Lactate (mmol/L) | 3.5 [2.7-5.4] |
| CRT (sec) | 5 [3-6] |
| ScvO2 (%) | 73 [65-79] |
| Delta pCO2(v-a) | 7 [5-10] |
| SOFA at 24-hours | 8 [5-11] |
| Renal replacement therapy | 17 (72) |
| Mechanical ventilation | 76 (322) |
| ICU length of stay (days) | 6 [3-12] |
| 28-day mortality | 166 (39) |

Data are presented as median [IQR 25-75] or count (percentage).

Definition of abbreviation: APACHE II: Acute Physiology And Chronic Health Evaluation II; SOFA: Sequential organ failure Assessment score;; ICU: Intensive care unit ;MAP: Mean arterial pressure, CVP: Central venous pressure; CRT: Capillary refill time. ; ScvO2: central venous oxygen saturation; Delta pCO2(v-a): difference between central venous carbon dioxide pressure and arterial carbon dioxide pressure
