# Supplementary material for: A lactate-targeted resuscitation strategy may be associated with higher mortality in patients with septic shock and normal capillary refill time: a post hoc analysis of the ANDROMEDA-SHOCK study
Source: Ann Intensive Care. 2020 Aug 26;10:114. doi: 10.1186/s13613-020-00732-1 (PMC7450018; doi:10.1186/s13613-020-00732-1)
Supplement: Supplementary file 2 — Additional File 2: Demographic, perfusion and hemodynamic characteristics and clinical outcomes of patients according to CRT status at T0 and T2. [file 13613_2020_732_MOESM2_ESM.docx]

Additional File 2: Demographic, perfusion and hemodynamic characteristics and clinical outcomes of patients according to CRT status at T_0_ and T_2_.

|  | **Normal-Normal** | **Normal-Abnormal** | **Abnormal-Normal** | **Abnormal-Abnormal** | **P value** |
| --- | --- | --- | --- | --- | --- |
| N° | 23 (88) | 3 (11) | 26 (96) | 48 (183) |  |
| Age (years) | 65 [46-72] | 69 [55-75] | 62 [49-72] | 69 [56-77] | 0.008 |
| Sex (female) | 46 (52) | 6 (54) | 42 (44) | 87 (48) |  |
| APACHE score | 18 [13-23] | 25 [19-33] | 21 [17-26] | 24 [18-29] | 0.0001 |
| SOFA score | 8 [6-10] | 9 [8-14] | 9 [7-12] | 10 [8-13] | 0.0001 |
| Sepsis origin | Abdominal 25 (28)  Pulmonary 28 (32)  Urinary 20 (23) Other 15 (17) | Abdominal 3 (27)  Pulmonary 5 (46)  Urinary 2 (18)  Other 1 (9) | Abdominal 39 (41)  Pulmonary 27 (28)  Urinary 17 (18)  Other 13 (14) | Abdominal 71 (39)  Pulmonary 47 (26)  Urinary 43 (23)  Other 22 (12) | 0.4 |
| MAP (mmHg) | 70 [65-83] | 64 [56-72] | 66 [61-76] | 64 [56-75] | 0.36 |
| CVP (mmHg) | 8 [5-12] | 10 [9-15] | 10 [6-14] | 9 [6-13] | 0.34 |
| Pre-protocol fluids (ml) | 2000 [1500-3000] | 2386 [1500-2500] | 2000 [1050-2500] | 2000 [1195-2500] | 0.161 |
| NE dose (mcg/kg/min) | 0.14 [0.08-0.25] | 0.12 [0.09-0.22] | 0.18 [0.11-0.29] | 0.29 [0.15-0.44] | 0.0001 |
| Lactate (mmol/L) | 3.0 [2.4-4.2] | 3 [2.6-4] | 3.5 [2.7-4.4] | 4.2 [2.9-6.5] | 0.0001 |
| CRT (sec) | 2 [2-3] | [2 -3] | 5 [4-6] | 6 [5-7] | 0.0001 |
| ScvO2 (%) | 76 [69-82] | 70 [63-79] | 70 [60-78] | 72 [62-78] | 0.001 |
| Delta pCO2(v-a) | 7 [4-9] | 7 [5-8] | 7 [4-10] | 7 [5-10] | 0.43 |
| Fluids administered in  boluses between 0-8  hours (ml) | 500 [0-1000] | 1500 [500-1900] | 1000 [500-2000] | 1500 [500-2500] | 0.0001 |
| Fluid balance at 8-h  hours (ml) | 1090 [319-1999] | 1700 [1014-2685] | 1211 [405-2368] | 1847 [980-3035] | 0.03 |
| SOFA 24 h | 7 [4-10] | 10 [7-10] | 8 [4-11] | 10 [6-13] | 0.0001 |
| Vasopressor test | 24 (27) | 3 (27) | 31 (32) | 73 (40) | 0.01 |
| Inodilator test) | 9 (10) | 2 (18) | 7 (7) | 43 (23) | 0.01 |
| ICU LOS | 7 [4-13] | 5 [2-14] | 6 [3-11] | 6 [2-13] | 0.58 |
| 28-days mortality | 20 (23) | 7 (63) | 35 (36) | 82 (45) | 0.001 |

Data are presented as median [IQR 25-75] or count (percentage). In some variables, data is presented with decimals due to their nature.

Definition of abbreviations: CRT: Capillary refill time; APACHE II: Acute Physiology And Chronic Health Evaluation II; SOFA: Sequential organ failure Assessment score; ICU: Intensive care unit ;MAP: Mean arterial pressure, CVP: central venous pressure; NE: norepinephrine; S_cv_O_2_: central venous oxygen saturation; Delta pCO_2_(v-a): difference between central venous carbon dioxide pressure and arterial carbon dioxide pressure.
