# Supplementary material for: A lactate-targeted resuscitation strategy may be associated with higher mortality in patients with septic shock and normal capillary refill time: a post hoc analysis of the ANDROMEDA-SHOCK study
Source: Ann Intensive Care. 2020 Aug 26;10:114. doi: 10.1186/s13613-020-00732-1 (PMC7450018; doi:10.1186/s13613-020-00732-1)
Supplement: Supplementary file 3 — Additional File 3: Clinical and interventions comparison between CRT non-responders at T2, according to study group. [file 13613_2020_732_MOESM3_ESM.docx]

**Additional file 3: Clinical and interventions comparison between CRT non-responders at T_2_, according to study group**

|  | **CRT abnormal at 2-hours**  **(n = 194)** | |  |
| --- | --- | --- | --- |
| Original study arm | CRT | Lactate | P |
| Number of patients | 102 (53) | 92 (47) |  |
| Age (years) | 69 [57-77] | 69 [55-78] | 0.90 |
| Sex | Female 55 (53)  Male 47 (47) | Female 38 (41)  Male 55 (59) | 0.08 |
| APACHE II score | 25 [18-30] | 24 [19-28] | 0.70 |
| SOFA score | 10 [8-13] | 10 [8-13] | 0.80 |
| Charlson Index | 3 [2-5] | 3 [1-5] | 0.45 |
| Sepsis origin n (%) | Urinary: 24 (24)  Pulmonary: 28 (27)  Abdominal: 36 (35)  Other: 14 (14) | Urinary: 21 (23)  Pulmonary: 24 (26)  Abdominal: 38 (41)  Other: 9 (10) | 0.40 |
| MAP (mmHg) | 65 [56-72] | 64 [56-76] | 0.73 |
| CVP (mmHg) | 9 [6-13] | 10 [7-13] | 0.61 |
| Pre-protocol fluids (ml) | 2000 [1000-2500] | 2000 [1425-3000] | 0.15 |
| NE dose (mcg/kg/min) | 0.3 [0.15-0.48] | 0.22 [0.11-0.4] | 0.12 |
| Baseline lactate (mmol/L) | 4 [2.9-6.3] | 4.2 [2.9-6.8] | 0.60 |
| Baseline CRT (s) | 6 [5-7] | 6 [4-7] | 0.78 |
| Baseline mottling Score | 1 [0-2] | 0 [0-2] | 0.30 |
| Baseline ScvO_2_ (%) | 72 [62-79] | 72 [63-78] | 0.99 |
| Baseline dCO_2_(v-a) | 7 [5-10] | 8 [6-10] | 0.31 |
| Fluid bolus 0-8h (ml) | 1500 [500-2500] | 1000 [400-2000] | 0.049 |
| Fluid balance 8h (ml) | 1700 [1014-2685] | 1995 [921-3100] | 0.70 |
| Vasopressor test | 40 (39) | 36 (39) | 0.90 |
| Inodilator test | 27 (26) | 18 (16) | 0.30 |
| Resuscitative interventions | 4 [2-6] | 3 [1-4] | 0.033 |
| SOFA 24 h | 10 [7-13] | 10 [6-13] | 0.90 |
| dSOFA 0-24 h | 0 [-2 -3] | 1 [-2 -3] | 0.50 |
| RRT | 18 (18) | 21 (22) | 0.40 |
| MV | 87 (85) | 76 (83) | 0.61 |
| ICU length of stay (days) | 6 [2-13] | 6 [2-13] | 0.56 |
| 28-day mortality | 47 (46) | 42 (45) | 0.95 |

Data are presented as median [IQ 25-75] or count (percentage).

Definition of abbreviation: CRT: Capillary refill time; APACHE II: Acute Physiology And Chronic Health Evaluation II; SOFA: Sequential organ failure Assessment score; ICU: Intensive care unit ;MAP: Mean arterial pressure, CVP: central venous pressure; NE: norepinephrine; ScvO2: central venous oxygen saturation; Delta pCO2(v-a): difference between central venous carbon dioxide pressure and arterial carbon dioxide pressure; dSOFA: delta SOFA; RRT: renal replacement therapy; MV: mechanical ventilation.
